# Predictors of poor control in adults with bronchial asthma

**DOI:** 10.6026/973206300221156

**Published:** 2026-02-28

**Authors:** Hemant Kumar Jain, Jitendra Kanjolia, Pavitra Chouhan, Shivani Parihar

**Affiliations:** 1 Department of General Medicine, Government Medical College, Datia, Madhya Pradesh, India; 2 Department of Pathology, Chirayu Medical College, Bhopal, Madhya Pradesh, India

**Keywords:** Asthma, adult, asthma control test, predictors, poor control, spirometry

## Abstract

Poor asthma control in adults remains a major challenge, leading to increased morbidity and healthcare usage. Therefore, it is of interest to identify clinical, demographic and behavioral predictors of poor asthma control in 120 adult patients. The results revealed that factors such as smoking, poor medication adherence, high BMI and lower lung function were significant predictors. Multivariate analysis highlighted key modifiable and non-modifiable factors contributing to poor control. This research advances knowledge by emphasizing the importance of early identification and targeted management of these predictors to improve asthma outcomes.

## Background:

Bronchial asthma is a chronic inflammatory airway disease characterized by variable airflow limitation and recurrent respiratory symptoms such as wheezing, shortness of breath, chest tightness and cough. Despite available therapeutic options and evidence-based guidelines, many adults with asthma continue to experience suboptimal control, which contributes to frequent symptoms, increased risk of exacerbations, reduced quality of life and greater healthcare utilization [1, 2]. Multiple demographic and clinical factors have been associated with poor asthma control in adult populations. Tobacco smoking has been repeatedly identified as a major risk factor for inadequate asthma control, as it exacerbates airway inflammation, accelerates decline in lung function and reduces responsiveness to anti-inflammatory therapy [1, 3]. Obesity is another important modifiable risk factor; higher body mass index (BMI) has been linked with a greater likelihood of uncontrolled asthma, more severe symptoms and reduced spirometric measures in adults [4, 5]. Impaired lung function itself, particularly lower forced expiratory volume in one second (FEV_1_), has been shown to correlate with poorer control and increased disease burden [1, 3]. Beyond these physiological determinants, behavioral and treatment-related factors such as poor medication adherence, incorrect inhaler technique and the presence of comorbidities, including allergic rhinitis, have been associated with uncontrolled asthma outcomes. Poor adherence to controller therapy is common and significantly compromises the achievement of asthma control targets [6]. Comorbid conditions like allergic rhinitis and other chronic inflammatory disorders frequently coexist with asthma and have been linked with worse control, likely due to overlapping inflammatory pathways and increased symptom burden [7]. Therefore, it is of interest to describe the clinical, demographic and behavioral predictors of poor asthma control in adult patients attending our clinic.

## Methodology:

This cross-sectional observational study was conducted at Dr. Hemant Jain clinic, Vidhya Vihar colony, Datia, Madhya Pradesh, India. The study aimed to identify clinical, demographic and behavioral predictors associated with poor asthma control in adult patients. Written informed consent was obtained from all participants prior to inclusion. Adults aged 18-65 years with a confirmed diagnosis of bronchial asthma according to the Global Initiative for Asthma (GINA) 2023 criteria were considered for inclusion. Patients with chronic obstructive pulmonary disease (COPD), interstitial lung disease, cardiac asthma, or other significant comorbid respiratory disorders were excluded. Pregnant and lactating women were also excluded to avoid confounding factors related to physiological changes in pregnancy. Based on previous prevalence studies indicates that approximately 40% of adult asthmatic patients' exhibit poor control [8] and assuming a 95% confidence interval with a 10% margin of error, the calculated minimum sample size was 92. To account for potential non-response or incomplete data, 120 patients were enrolled consecutively using a convenience sampling approach. Demographic data (age, sex, body mass index [BMI], educational status, occupation), clinical history (duration of asthma, comorbidities, smoking status, medication adherence) and environmental exposures (allergens, occupational irritants) were collected using a structured pretested questionnaire. Asthma control was assessed using the validated Asthma Control Test (ACT) questionnaire.

Patients scoring ≤19 were classified as having poorly controlled asthma, while those scoring ≥20 were considered well-controlled. Spirometry was performed using a standardized portable spirometer (Model: [Specify Model], Manufacturer: [Specify]) following American Thoracic Society (ATS) guidelines. Forced expiratory volume in 1 second (FEV_1_), forced vital capacity (FVC) and FEV_1_/ FVC ratio were recorded. Baseline and post-bronchodilator readings were noted. Medication adherence was evaluated using the Morisky Medication Adherence Scale-8 (MMAS-8) and inhaler technique was assessed by a trained respiratory therapist using a standardized checklist. Patients demonstrating incorrect technique received on-site correction and education. Data were entered into Microsoft Excel and analyzed using SPSS version 26.0. Continuous variables were expressed as mean ± standard deviation (SD) and categorical variables as frequencies and percentages. The association between potential predictors and asthma control was analyzed using chi-square test or Fisher's exact test for categorical variables and independent t-test for continuous variables. Variables with p < 0.10 in univariate analysis were entered into a multivariate logistic regression model to identify independent predictors of poor asthma control. A p-value <0.05 was considered statistically significant.

## Results:

A total of 120 adult patients with bronchial asthma were included in the study. The majority of participants were middle-aged, with a slight predominance of males. Most patients had a BMI in the overweight range and approximately half had a history of smoking. Comorbid conditions, particularly allergic rhinitis, were common among the cohort (Table 1). Spirometric assessment revealed that nearly one-third of patients had an FEV below 70% of predicted values, indicative of moderate airflow limitation. Based on ACT scores, poor asthma control was observed in 61.7% of participants, highlighting a substantial burden of uncontrolled disease in this population (Table 2). Poorly controlled asthma was more frequently associated with longer disease duration, higher BMI, smoking, comorbid allergic rhinitis and reduced lung function. Multivariate logistic regression identified several independent predictors of poor asthma control (Table 3). Reduced FEV (<70% predicted) emerged as the strongest predictor, conferring more than a fourfold increase in risk. Poor adherence to prescribed medications was similarly associated with a markedly elevated likelihood of uncontrolled asthma. Other significant contributors included current smoking, higher BMI and longer disease duration. Comorbid allergic rhinitis showed a borderline association with poor control. Age above 45 years demonstrated a trend toward increased risk but did not reach statistical significance.

## Discussion:

In this cross-sectional analysis of adult asthmatics, a substantial proportion of participants exhibited poor asthma control, with modifiable and non-modifiable factors contribute to this outcome. Consistent with other recent real-world investigations, reduced lung function emerged as a robust predictor of poor control. Lower FEV values have been repeatedly linked to uncontrolled asthma and exacerbations in clinic-based and population studies, highlighting the central role of airflow limitation in determining control status [9, 10]. Behavioral factors such as smoking and medication adherence were also independently associated with poor control in our population. Smoking influences airway inflammation and diminishes corticosteroid responsiveness, making asthma more difficult to manage; this has been confirmed in dedicated models of smoking asthmatics where smoking history and medication adherence predicted poor control outcomes [9]. Likewise, non- adherence to prescribed inhaler therapy has been identified as a common and critical determinant of uncontrolled asthma, reinforcing the need for interventions that improve adherence in routine care [11]. Obesity, another modifiable risk factor identified in our multivariate model, has been implicated in numerous studies as a factor that complicates asthma control. Excess adiposity affects respiratory mechanics and may exacerbate airway inflammation, contributing to worse asthma outcomes [12]. Comorbid conditions, including allergic rhinitis and other chronic diseases, also complicate management by overlapping symptomatology, increasing disease burden and impairing control, as demonstrated in large population databases and specialist registries where multimorbidity heightened the risk of poor asthma outcomes [10, 13]. Our findings align with this evidence by illustrating the influence of comorbid disease on control status. Advanced age was positively associated with poor control in univariate analyses, though its effect was attenuated in multivariate modeling. Nonetheless, age-related changes in physiology, immune function and comorbidity burden likely contribute to poorer outcomes in older asthmatics, as suggested in epidemiological studies of adult and elderly asthma cohorts [14]. Taken together, these observations emphasize the multifactorial nature of asthma control, where patient-related behaviors, physiological impairment and comorbidities must be addressed in comprehensive management strategies.

## Conclusion:

In adult patients with bronchial asthma, poor disease control is prevalent and is strongly influenced by both modifiable factors, including smoking, elevated BMI and suboptimal medication adherence, as well as non-modifiable factors such as longer disease duration and reduced lung functions. Targeted interventions addressing these predictors-through optimized pharmacotherapy, adherence reinforcement, lifestyle modification and early identification of high-risk individuals may significantly improve asthma outcomes and reduce morbidity in this population.

## Figures and Tables

**Table 1:** Baseline Demographic and clinical characteristics of study participants (n = 120)

**Characteristic**	**Total (n = 120)**	**Well-Controlled (n = 46)**	**Poorly Controlled (n = 74)**
Age (years), mean ± SD	42.1 ± 12.3	40.5 ± 11.8	43.2 ± 12.5
Male, n (%)	68 (56.7%)	28 (60.9%)	40 (54.1%)
BMI (kg/m^2^), mean ± SD	25.8 ± 3.6	24.9 ± 3.2	26.4 ± 3.8
Duration of asthma (years), mean ± SD	8.5 ± 5.1	6.9 ± 4.5	9.5 ± 5.2
**Smoking status, n (%)**			
Current smoker	32 (26.7%)	6 (13.0%)	26 (35.1%)
Ex-smoker	20 (16.7%)	8 (17.4%)	12 (16.2%)
Never smoked	68 (56.6%)	32 (69.6%)	36 (48.7%)
**Comorbidities, n (%)**			
Allergic rhinitis	42 (35%)	10 (21.7%)	32 (43.2%)
Diabetes mellitus	18 (15%)	4 (8.7%)	14 (18.9%)
Hypertension	24 (20%)	6 (13%)	18 (24.3%)
Spirometry FEV- (% predicted), mean ± SD	71.5 ± 12.8	82.2 ± 8.7	64.1 ± 10.5
ACT score, mean ± SD	17.8 ± 4.5	22.1 ± 1.8	14.6 ± 2.7

**Table 2:** Asthma control according to ACT Scores

**Asthma Control**	**n (%)**
Well-Controlled (ACT ≥20)	46 (38.3%)
Poorly Controlled (ACT ≤19)	74 (61.7%)

**Table 3:** Multivariate logistic regression analysis of predictors of poor asthma control

**Predictor**	**Adjusted Odds Ratio (OR)**	**95% Confidence Interval (CI)**	**p-value**
Age > 45 years	1.8	0.9 - 3.4	0.08
BMI ≥ 25 kg/m^2^	2.2	1.1 - 4.3	0.03*
Duration of asthma > 10 years	2.5	1.2 - 5.0	0.01*
Current smoker	3.1	1.4 - 6.7	0.004*
Comorbid allergic rhinitis	2	1.0 - 4.0	0.05
FEV < 70% predicted	4.3	2.0 - 9.2	<0.001*
Poor medication adherence (MMAS-8 < 6)	5.1	2.4 - 10.8	<0.001*
